# Scalable thermoelectric fibers for multifunctional textile-electronics

**DOI:** 10.1038/s41467-020-19867-7

**Published:** 2020-11-26

**Authors:** Tianpeng Ding, Kwok Hoe Chan, Yi Zhou, Xiao-Qiao Wang, Yin Cheng, Tongtao Li, Ghim Wei Ho

**Affiliations:** 1 Department of Electrical and Computer Engineering, National University of Singapore, 4 Engineering Drive 3, Singapore, 117583 Singapore; 2 Department of Materials Science and Engineering, National University of Singapore, 9 Engineering Drive 1, Singapore, 117575 Singapore; 3 Institute of Materials Research and Engineering, A*STAR (Agency for Science, Technology and Research), 3 Research Link, Singapore, 117602 Singapore

**Keywords:** Devices for energy harvesting, Thermoelectric devices and materials, Electronic devices, Electronic properties and materials, Synthesis and processing

## Abstract

Textile electronics are poised to revolutionize future wearable applications due to their wearing comfort and programmable nature. Many promising thermoelectric wearables have been extensively investigated for green energy harvesting and pervasive sensors connectivity. However, the practical applications of the TE textile are still hindered by the current laborious p/n junctions assembly of limited scale and mechanical compliance. Here we develop a gelation extrusion strategy that demonstrates the viability of digitalized manufacturing of continuous p/n TE fibers at high scalability and process efficiency. With such alternating p/n-type TE fibers, multifunctional textiles are successfully woven to realize energy harvesting on curved surface, multi-pixel touch panel for writing and communication. Moreover, modularized TE garments are worn on a robotic arm to fulfill diverse active and localized tasks. Such scalable TE fiber fabrication not only brings new inspiration for flexible devices, but also sets the stage for a wide implementation of multifunctional textile-electronics.

## Abstract (web summary)

Despite the potential of incorporating thermoelectric (TE) fibers into textile electronics for green energy harvesting, existing fabrication methods are not commercially viable. Here, the authors report a scalable gelation extrusion fabrication strategy for realizing alternating p/n-type TE fibers.

## Introduction

Advances in miniaturization and integration of electronics have made significant strides in wearable technology, such as electronic skin (e-skin) and textiles based electronics^1–5^. In particular, smart textiles comprising of functional fibers and/or integrated with microelectronic systems, are receiving increasing attention due to their conformality, breathability, and unobtrusiveness^6–8^. Through assembling different functional fibers or components, the smart textiles have capacity of sensing, communicating, actuating, energy harvesting, and so on^9–15^. Thermoelectric (TE) materials that can convert temperature gradient into an electrical voltage (Seebeck effect) or vice versa (Peltier effect) are particularly attractive because of their versatility in wearable electronics that perform touch sensing, health monitoring, personal temperature regulation, and body heat energy harvesting^14,16–22^.

However, existing commercial TE materials are rigid, heavy, and brittle, generally fabricated into blocky architectures from costly materials via elaborate routes. This greatly constrains their widespread adoption in textile based wearable electronics^23–25^. Despite intensive research efforts and advances in textile based TE devices utilizing bismuth telluride^26^, carbon nanotube (CNT)^27^, poly(3,4-ethylenedioxythiophene) (PEDOT)^28^, or polyaniline^29^, fabrics comprised of TE are only at the early stage and far from practical implementation, largely due to unavailability of industrially scalable and cost-effective fabrication techniques. Notably, most wearable TE based textiles are realized by coating regular fibers with TE sheaths^26^, filling TE materials at the interspace of the fabrics^30^ or incorporating of other fibers to form yarns^31^, which will inevitable lower space efficiency and fray/wear out with extended mechanical friction and deformation induced by body movement, resulting in unstable and attenuated performance. Given the usual wearable operating temperature (lower than 100 ^o^C) and continual contact with skin, the organic TE materials are especially suitable for textile electronics due to the advantages of earth-abundance, nontoxicity, light-weight, and ease of synthesis^29,32^. Additionally, to facilitate subsequent weaving into textiles and to improve the performance efficiency, the p-type and n-type TE elements, in general, are thermally integrated in parallel but electrically connected in series, which further aggravate the difficulty of continuous large scale fabrication of TE textile electronics. Currently, there are still few, if any, practical manufacturing methods to directly fabricate TE fibers with seamless p/n junctions composed garment capable of multitasking.

Here, we propose a colloidal gelation extrusion of TE fibers, which demonstrates simplicity, controllability, and industrial scalability manufacturing of mechanically robust and flexible TE fibers. Taking the advantages of hydrocolloid constituted network and its rheological property, superior confinement of heterogeneous molecular particles within the continuous matrix is particularly apt to produce high homogeneity, good interface bonding and alternating p/n-type segments. Such axially-aligned p/n-type TE fibers dramatically reduce the complexity of the subsequent integration in textile electronics, which also provide additional possibilities for multifunctional configurability. Through weaving of the alternating p/n-type TE fibers into fabrics, TE textiles can perform diverse functions, including conformal heat energy harvesting cloth, localized touch panel for display and light orientation sensing for communication. Furthermore, the clothes worn on a robotic arm demonstrates different functionalities, rendering it capable of body heat energy harvesting, phototaxis and temperature reflexivity. This work is promising in delivering a self-powered multi-purpose textile with built-in intelligence that responds to external changes/environments.

## Results

### Fabrication of alternating p/n-type TE fibers

**Fig. 1:**
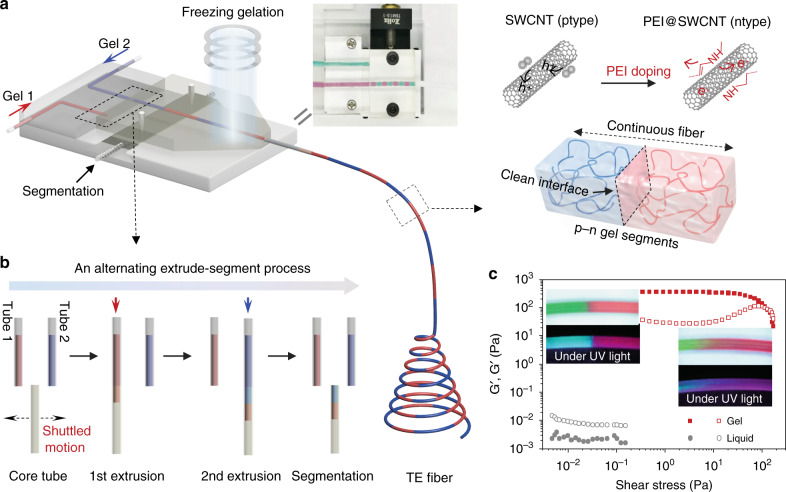
Colloidal gelation extrusion process. **a** Schematic of the continuously alternating extrusion process. Hydrocolloidal network confines heterogeneous molecular particles well in the matrix to form an alternating p/n-type TE fiber. **b** Sketch of the automatic extrude-segment assembly line. **c** Shear storage moduli G’ and loss moduli G” as a function of shear stress for PVA compounds. The solid and hollow symbols represent G’ and loss moduli G”, respectively. Insets: photos of the alternatively extruding samples using PVA-based compounds in liquid (bottom right) and hydrocolloid (top left) forms.

The flexible TE fibers consisting of single-walled carbon nanotubes (SWCNTs) (Supplementary Fig. 1 and Supplementary Fig. 2) and polyvinyl alcohol (PVA) hydrocolloids were fabricated through a continuously alternating extrusion process (Fig. 1a, Supplementary Movie 1). The room temperature extrusion is user-programmed and computer-controlled to automate an extrude-segment assembly line (Fig. 1b). The p-type and n-type composite gel in the two separate polytetrafluoroethylene (PTFE) tubes move back and forth to closely align and extrude into a core tube. Both the loss modulus G” and storage modulus G’ of the formulated hydrocolloids are prepared to be very low with G’ < G”, enabling the gel deformable under applied pressure^33^. The rheological properties of hydrogel after freezing gelation show much higher G’ and G” of around three orders of magnitude increase. The G’ of the gel is higher than the G” below the critical shear stress point, meaning that the gel will maintain its shape as long as the shear stress is lower than the critical value^34^. Apart from the versatile tunability of the hydrocolloids, another notable advantage is that the migration of the solvent is severely restricted by the PVA polymer networks. Hence, alternatingly extruded gels are shown to preserve the p-n junctions and exhibit fairly clear interfaces even under continual compressive and shear stresses along the core tube (Fig. 1c, top inset). In comparison, untailored solid and liquid matrixes face the issue of cross-mixing or even blending when they are successively extruded into a single tube (Fig. 1c, bottom inset).

As indicated by the rheology properties evolution, the SWCNT/PVA and polyethyleneimine (PEI) doped compounds exhibit a dramatic decrease in fluidity (Supplementary Fig. 3), similar to the PVA hydrogel. This aids in the restriction of the constituents i.e., SWCNTs and dopants within the specific segments of TE fiber (Supplementary Fig. 4). Moreover, the PVA-based hydrogel has the ability to adhere and heal when subjected to a freezing gelation process to realize firm and close contact between the adjacent gel segments. Supplementary Fig. 5 shows as-prepared alternating PVA gels with different user-defined segment lengths.

### Mechanical and TE properties of TE fibers

**Fig. 2:**
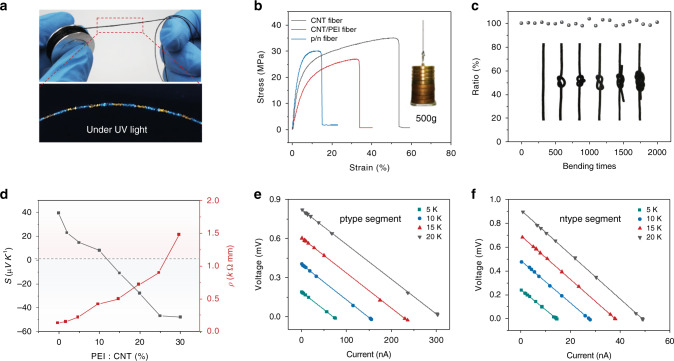
Scalable TE fibers of high strength and flexibility. **a** Photographs of the manufactured TE fiber under natural (upper) and UV lighting (lower). **b** Stress-strain curves of different types of SWCNT/PVA TE fibers. Inset: photo of a single TE fibers sustaining 500 g weight. **c** Resistance changes of a TE fiber under continuous bending. Inset: the photos of different TE-fiber knots. **d** The Seebeck coefficient and electrical conductivity vary with different PEI and CNT weight ratios. **e**, **f** TE performances of single pure (e) p-type and (f) n-type fibers.

With the bypassing of the traditional arduous synthesis and assembly of p/n-type TE fiber of limited scale, the proposed alternating extrude-segment assembly line demonstrates the feasibility of digitalized manufacturing of continuous meter-scale TE fibers (Fig. 2a). presents a TE fiber under UV light which reveals an alternately distributed yellow (p-type) and green (n-type) TE segments. Supplementary Fig. 6 shows the profile of the TE fiber, exhibiting a relatively uniform diameter and evenly distributed CNTs. Moreover, PVA-based composites have been demonstrated to be of high strength and toughness^35,36^. Similarly, the as-prepared TE fibers also possess inherent mechanical merits as the TE fibers with or without PEI present high tensile strength over 20 MPa (Fig. 2b). Notably, this signifies that the bonding between the adjacent p/n-type segments is very strong and able to hold weights, as heavy as 500 g (Fig. 2b, inset). As a weaving material, the flexibility of the TE fiber is an essential requirement for body conformation, comfort and aesthetics. To verify its flexibility, the fiber was knotted into different styles or with other fiber (Fig. 2c, inset). Moreover, the resistance changes under continuous bending of a TE fiber were recorded in Fig. 2c to demonstrate its satisfactory electrical robustness.

In order to switch the majority carriers in the SWCNTs from holes to electrons, sufficient amine-rich PEI molecules should be added to inject ample electrons into the SWCNTs. Thus, the weight ratio of the SWCNTs and PEI profoundly affects the properties of the TE fiber. Figure 2d presents the Seebeck coefficient (*S*) and electrical resistivity (*ρ*) change as the weight ratio of the PEI: SWCNTs increase gradually (the weight ratio of SWCNTs:PVA is fixed as 1:2). The pristine SWCNTs/PVA composite without dopant shows a positive Seebeck coefficient (39.5 µV K^−1^), indicating the composite is p-type. As the PEI content increases, the Seebeck coefficient gradually shifts to negative (−48 µV K^−1^ at PEI: SWCNTs weight ratio of 30%), which indicates the switching to n-type. The resistance of the composite increases with the PEI content. Since high Seebeck coefficient facilitates the increase of temperature gradient induced TE voltage while high resistance results in low current, the weight ratio of PEI and SWCNTs is chosen to be 25% to ensure a minimal tradeoff between the TE voltage and current. Figure 2e, f shows the voltage-current varying at different temperature differences for 10 mm long p-type and n-type TE fibers, respectively. It can be seen that both the voltage and current increase linearly with the temperature differences. Supplementary Fig. 7 shows the TE voltages of the p-type and n-type TE fibers vary with the temperature difference.

### Conformal heat energy harvesting of TE textiles

**Fig. 3:**
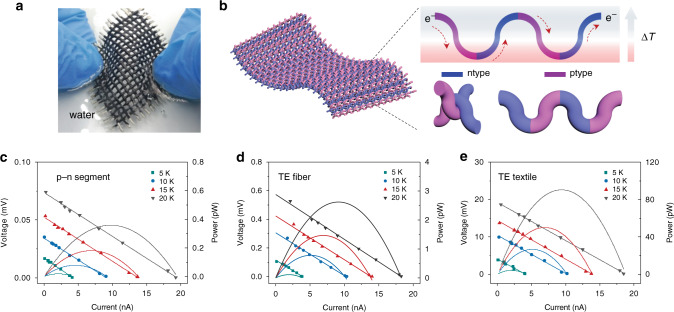
Flexible TE textile for conformable heat energy harvesting. **a** A weaved TE textile with waterproof layer washed in water. **b** Schematic of a TE textile harvesting thermal energy in an out-of-plane direction. The pitch waves correspond the segment length to realize successive p-n junctions alternate between hot and cold surfaces. The carriers flow in same direction along the fiber to multiply the voltage. **c**–**e** TE performance of a single p/n TE pair, a single fiber with 8 p/n TE pair and a TE textile weaving by 33 TE fibers in out-of-plane direction under different temperature differences between the substrate and the ambient.

Alternating p/n-type segmented TE fibers were plain weaved into flexible TE textile. Moreover, the TE textiles can be processed to be of different colors for aesthetic purpose and/or coated with other materials to be waterproof and washable as shown in Fig. 3a and Supplementary Movie 2. The p/n-type units are electrically connected in series, similar to the commercial TE generators, which have been demonstrated to be an efficient design (Fig. 3b). In this configuration, the same designated p/n-type length were used to ensure not only the pitch weave matches the segment but also to ascertain successive p-n junctions alternate between hot and cold surfaces. Consequently, the carriers in each TE units will flow in same direction along the fiber, so that voltage multiplication can be achieved. Depending on different connections between the terminals of each fiber, on-demand voltage or current multiplication can be achieved by series or parallel electrical connections.

The heat flow direction for such in-plane configured TE textile is in thickness direction, different from conventional flexible TE generators that harvest heat in an in-plane direction. As the textile is directly made by weaving the TE fibers (diameter of only 0.8 mm, length of p/n-type segment is around 2 mm) without incorporating other yarns, the thickness of the textile is quite small, leading to a small temperature gradient and hence a modest TE performance. For a single p/n TE pair, both the voltage and current monotonically increase as the temperature of the substrate increases from 5 to 20 °C higher than the ambient (Fig. 3c). The temperature difference between the top and bottom of the textile is calculated to be around 1 °C at 20 °C, which is more than one order magnitude lower than the recorded one.

For a TE fiber composed of 8 pairs of p/n couples, the open-circuit voltage approximately amplified proportionally, for instance, the voltage increases from ~0.12 to ~0.52 mV at same apparent temperature difference of 20 °C (Fig. 3d). Meantime, the TE currents are almost same at corresponding temperature difference, agreeing with the tandem electrical circuit. We also develop a finite element model that allows us to calculate the temperature and electrical potential distributions along the fiber (detail is described Supplementary Note 1). The simulated results correspond well to the experiments (Supplementary Fig. 8). Further, the heat energy harvesting ability on curve surface was conceptually demonstrated by wrapping a piece of cloth composed of 33 TE fibers on a filled with water at different temperature (Supplementary Fig. 9a). The TE fibers are woven in plain with in-series electrical connection at the terminals. As expected, the voltages are amplified by around 33-fold compared with a single fiber, while the current remains the same (Fig. 3e, Supplementary Fig. 9b). This demonstration not only shows the multiplication effect of the energy harvesting ability from one-dimensional fiber to two-dimensional textile, but also proves the conformability of the textile to a nonplanar surface.

### TE textiles for heat and light sensing

**Fig. 4:**
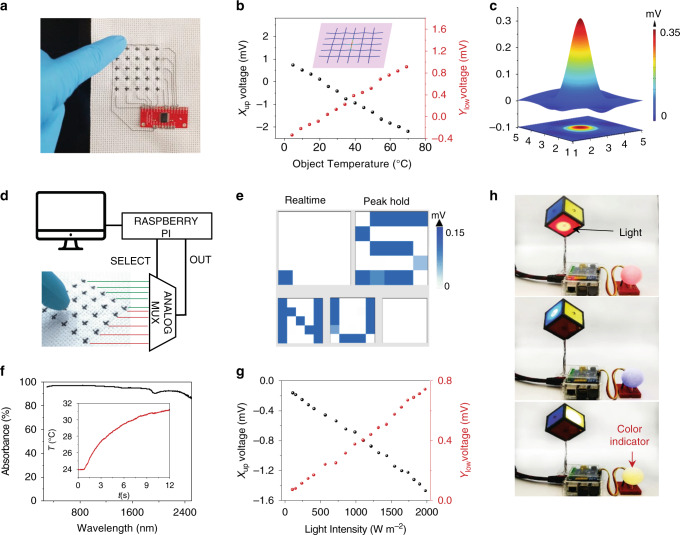
TE textile for touch panel and communication. **a** Photograph of a 5 × 5 pixels touch panel with cross-stitched TE fibers. **b** Signals of the TE fibers when the node is contacted with different object temperatures. Inset: simulated temperature distribution of the (3,3) node in contact with 50 °C object. **c** The resulting signal of the panel when touched by a finger at (3,3) node. **d** Circuit diagram of the touch panel. **e** Photo of a “NUS” writing on the touch panel. **f** The absorption spectrum of the SWCNT/PVA fiber. Inset: temperature evolution of the fiber when irradiated by a beam of light with 1000 W/m^2^. **g** The signals of the TE fibers when the node is irradiated by light with different intensities. **h** Light communication demonstration that sense the incident light direction.

Taking the advantage of the continuous p-type and n-type TE fiber, 10 TE fibers are easily woven into a patch of cross-stitch, as shown in Fig. 4a. Every fiber composes of three p/n pairs with each p/n segment of 10 mm in length. All of the fibers cross at the p/n joints, as shown in Supplementary Fig. 10a. When a specific joint node contacts a hot object, the contact point displays a much higher temperature than the adjacent node as simulated in the inset in Fig. 4b (detail is described in Supplementary Note 1). Because of the enlarged segment length and well thermal insulated cotton cross-stitch yarns, the adjacent node around the contact point stay at a constant temperature. A touch induced temperature gradient along the p/n TE fiber manifests an electrical potential difference between the terminals of the contacted fiber. Conversely, the non-contacted fibers do not generate electrical signals (Supplementary Fig. 10b, Supplementary Note 1).

To locate the geometry coordinates, the warp and weft TE fibers are *x*- and *y*-axes defined. With an ambient temperature of 22 °C, the signal intensities when contacted with different temperatures were investigated (Fig. 4b). As the object temperatures increase from 5 to 70 °C, the voltage along the x axis decreases from 0.85 to −2 mV. Meantime, the voltage along the *y*-axis, which is underneath the direct contacted fiber, indicates a relatively small change from −0.3 to 1.0 mV. Both the correspondences between the voltage signals and the object temperatures are stable and nearly linear, laying the foundations for touch positioning. Figure 4c and Supplementary Fig. 11a, b show the signals of each fibers when node (3,3) was touched by a finger. The contacted X-3 and Y-3 fibers generate a strong signal as opposed to the absence of signal for the non-contact ones. The signal-to-noise ratio was calculated (Supplementary Fig. 11c-d), revealing a well discerning capability. The position addressable ability of this panel is also verified (Supplementary Fig. 12). With the 5 × 5 pixels touch panel, we further realize a hand-writing alphabetical ‘NUS’ inputs (Fig. 4d, e, Supplementary Note 2 and Supplementary Movie 3).

Benefitting from the superior light absorbing ability of carbon material (Fig. 4f), the fibers easily heat up when irradiated by light. Inset in Fig. 4f shows the temperature evolution of a fiber when irradiated by a light beam of 1000 W m^−2^. The photothermal heat will induce a temperature gradient between the light exposed and nonexposed regions to generate electrical signals (Fig. 4g). Utilizing the photothermal light sensing property, TE fibers on the six faces of a cube was constructed and successfully realized light communication to accurately perceive the incident light orientation (Fig. 4h, Supplementary Note 2 and Supplementary Movie 4).

### Modularized TE textiles for multitasking robot

**Fig. 5:**
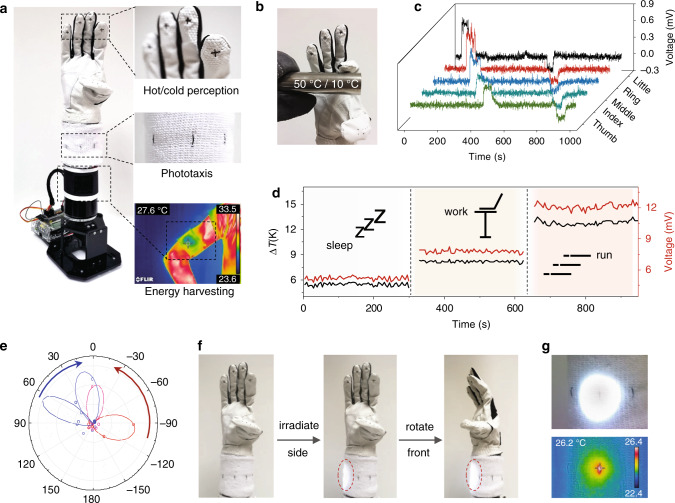
A robotic arm wearing modularized TE garments for multiple tasks. **a** Photograph of a robotic arm wearing multifunctional TE textiles for temperature perception (hand), phototaxis (wrist), and energy harvesting (arm). **b** Photo of temperature perception of the finger touching a hot/cold metal rod. **c** TE signals of the five fingers when the hand separately contacts with a hot and cold object. **d** Energy harvesting of a TE cloth worn on the arm at different states. **e** Signals of the phototaxis wrist band when irradiated with light beam at different orientations. **f** Photos for arm rotated to track the light beam. **g** Enlarged photo (upper) and IR image (lower) of TE node when irradiated by a light beam.

The TE fiber composed garments can provide modularized solutions to equip the wearer with various functions, offering a strategy for future conformal robotic electronics. Figure 5a shows a robotic arm capable of hot/cold perception, phototaxis and energy harvesting rendered by TE-fiber glove, band wrist and sleeve, respectively (details are described in Supplementary Note 2). With a minimal pair of a single crossed p/n TE fiber stitched at the glove fingertips, the robotic arm can sense an object’s temperature and adaptively grasp or loosen its grip accordingly (Fig. 5b, c). The phototaxis demonstration was realized by eight TE fibers, evenly distributed around a wristband. When a light beam shines from a certain orientation, the particular irradiated fiber will generate a TE signal which in turn gives an electrical cue to rotate the arm to face the light source (Fig. 5e–g). Supplementary Movie 5 shows the feedback control of a robotic arm wearing TE garments including reflex of hot/cold subject and phototaxis. To manifest the body heat harvesting, a TE cloth was worn on a person’s arm. The local body surface temperatures at different states i.e. sleeping, working and exercising generate different TE voltages of 5, 7, and 12 mV, respectively (Fig. 5d). Such conformable body heat harvesting textile can also be scaled up to be integrated into other wearable self-powering systems to charge batteries or power electronics directly. With such a suit of TE textiles, the garment enables a robot to multitasking, not limited to, sensing and energy harvesting.

## Discussion

In summary, we develop a scalable gelation extrusion of SWCNT/PVA TE fibers of p-type and n-type segments that are successively integrated into a continuous fiber. On the basis of these scaled up p/n-type TE fibers, textiles of different configurations are synthesized to realize a variety of functions, including energy harvesting on curved surface, multi-pixel touch panel for hand writing and communication. In addition, through wearing different modularized TE textiles, multiple situational reflex and self-powering capabilities are successfully endowed to a robotic arm. It is worth noting that this scalable fabrication strategy is also suitable for other TE materials and polymer matrix. This work not only demonstrates a promising approach towards scalable fabrication of TE textiles, but also reveals the prospect of a generation high tech textile-electronics.

## Methods

### Preparation of extruding gels

For PVA gels preparation: First, PVA powder (M_w_ 89,000–98,000, 99 + % hydrolyzed, Sigma–Aldrich) was mixed in DI water with 10 wt% under vigorous stirring at 85 °C until the solution became clear. Then, the as-prepared PVA solution was separately mixed with the green and red water-soluble food dyes (Bake King Company), forming the green and red PVA solution, respectively. Next, the two dyed solutions were separately extruded into two PTFE tubes (inner diameter = 1.6 mm, outer diameter = 2 mm). They were frozen for 3 h at around −20 °C to form hydrocolloid. For SWCNT/PVA gels preparation: Firstly, 500 mg SWCNT powders (60% purity, Nano Solutions Inc.), 100 mg sodium dodecyl sulfonate (SDS, 98.5%, Sigma–Aldrich), 10 g PVA solution (10 wt% in DI water) were added into 250 ml DI water, and then the mixture was probe sonicated for 20 min to uniformly disperse the SWCNTs in the solution. Subsequently, the mixture was concentrated to 15 g by heating and stirring. Next, the concentrated mixture was divided in half and separately mixed with yellow and green UV fluorescents (ZnS particles doped with different elements, Yaodexing Technology Inc.). To make the PEI doped SWCNTs, branched PEI (M.W. 600, Sigma–Aldrich) with certain weight was mixed into the concentrated mixture (adding 20 mg green UV fluorescent) and then heated at 80 °C in nitrogen atmosphere for 6 h. Then the SWCNT/PVA mixture (adding with green UV florescent) and the PEI doped SWCNT/PVA mixture (adding 20 mg yellow UV florescent) were separately extruded into two PTFE tubes (inner diameter = 1.6 mm, outer diameter = 2 mm). After frozen at −20 °C for 6 h and defrosting, the SWCNT/PVA gels were formed.

### Fabrication of p/n-type alternating fibers

Two PTFE tubes containing as-prepared gels (i.e., core tubes) were closely fixed in the two home-made acrylic channels and the other ends were connected with two syringes pumps. Another PTFE tube (inner diameter = 1.6 mm, i.e., core tube) fixed in an acrylic mold was moved repeatedly along the track by a linear motor. When the core tube was moved to align to one inner tube, a certain amount of contained gel was extruded into the core tube; then the core tube linearly moved to align to another inner tube to fill the gel extruded from the tube. The inner tubes and core tube were contacted very closely during processing. The gel would be segment apart by the closely contacted acrylic molds between the closely contacted inner tube and core tube. All the procedures were controlled by computer program as depicted in Fig. 1b, and this cycle repeats. When the two gels were alternatingly extruded into the core tube, the tube was then frozen for 6 h at −20 °C to heal the adjacent gel segments together. Then the alternating gel was extruded out and dried at 50 °C to get the alternating fibers. To coat waterproof layer on the TE fibers, the fibers were dipped in diluted UHU glue and naturally dried. This waterproof layer also served as electrical insulating layer. For the alternating colored PVA fibers or SWCNT/PVA-based TE fibers, the corresponding gels prepared previously were used to be extruded.

### Characterization and tests

The morphology of the fibers was characterized by SEM (FESEM, JEOL FEG JSM 7001 F). A MCR302 rheometer (Anton Paar) with cone-plate geometry was used to test the rheological property at 22 °C and oscillatory amplitude sweeps were performed at 1 Hz with strain values from 0.01 to 100%. The tensile stress-strain test was implemented using MTS Tytron 250 Microforce Testing System. Bending test was conducted with a home-made software controlled linear motor. The TE textile for energy harvesting was fabricated using the plain weave technique and the fibers were connected in series. Two K-type thermocouples were put in the water in the beaker and outside ambient to record the temperature of the beaker and ambient, respectively. The TE textiles for touch panel and light communication were prepared by weaving the TE fibers into a piece of cross-stich and hollow cube, respectively. Hot/cold perception glove and phototaxis wrist band were made by weaving TE fibers into the fingertips of a glove and a wrist band, respectively. The detailed controlling modules and circuit diagrams are described in Supplementary Note 2. Temperature was measured and recorded using NI-9212 Thermocouple Input module 5 s later after the samples were touched by objects or irradiated by light. The open-circuit voltage and short-circuit current of device were measured and recorded by nanovoltmeter (Keithley 2182 A) and electrometer (Keithley 6517B). The resistance of the fiber was tested by LCR meter (Tonghui TH2830). The infrared images were captured and recorded using the infrared camera (E50, FLIR Systems).

## Supplementary information

Supplementary Information

Supplementary Movie 1

Supplementary Movie 2

Supplementary Movie 3

Supplementary Movie 4

Supplementary Movie 5

Description of Additional Supplementary Files

## Data Availability

The authors declare that the data supporting the findings of this study are available within the paper and its Supplementary Information files. All other data are available from the authors upon reasonable request.
