# Supplementary material for: Scalable thermoelectric fibers for multifunctional textile-electronics
Source: Nat Commun. 2020 Nov 26;11:6006. doi: 10.1038/s41467-020-19867-7 (PMC7693281; doi:10.1038/s41467-020-19867-7)
Supplement: Supplementary file 1 — Supplementary Information [file 41467_2020_19867_MOESM1_ESM.pdf]

Supporting Information

Scalable thermoelectric fibers for multifunctional textile-electronics

Tianpeng Ding[1], Kwok Hoe Chan[1], Yi Zhou[1], Xiao-Qiao Wang[1], Yin Cheng[1], Tongtao Li[1] and Ghim Wei Ho[1,2,3]*

1. Department of Electrical and Computer Engineering, National University of Singapore, 4 Engineering Drive 3, Singapore 117583
2. Department of Materials Science and Engineering, National University of Singapore, 9 Engineering Drive 1, Singapore 117575
3. Institute of Materials Research and Engineering, A*STAR (Agency for Science, Technology and Research), 3 Research Link, Singapore 117602, Singapore.
*Correspondence: elehgw@nus.edu.sg (Ghim Wei Ho)

This supplement contains

Supplementary Figures 1-12

Supplementary Note 1: Thermal and electrical simulations

Supplementary Note 2: Controlling modules for the demonstrations

Supplementary Movies:
File Name: Supplementary Movie 1

Description: Alternating extrude-segment process.

File Name: Supplementary Movie 2

Description: Stability test of TE fabric with washing in water and rubbing.

File Name: Supplementary Movie 3

Description: Handwriting 'NUS' alphabet input.

File Name: Supplementary Movie 4

Description: Light communication device to perceive the incident light orientation.

File Name: Supplementary Movie 5

Description: Feedback control of robot arm wearing TE garments including reflex of hot/cold subject and phototaxis.

PRODUCT	DESCRIPTION	CARBONACEOUS PURITY*	METAL CONTENT wt% (from TGA in air)	PRICE	MINIMUM ORDER
AP-SWNT	As prepared	60 - 70%	< 30	$35/g	2 grams
P2-SWNT	Purified, low functionality	>90%	4-8	$280/g	0.5 grams
P3-SWNT	Purified, high functionality	>90%	5-7	$280/g	0.5 grams

Supplementary Figure 1 | Products description and price information of SWCNTs. These are acquired from the website of Carbon Solutions, Inc. Considering the huge disparities in prices, we choose the cheaper raw AP- SWCNTs with stated purity of 60 % to fabricate the TE fibers

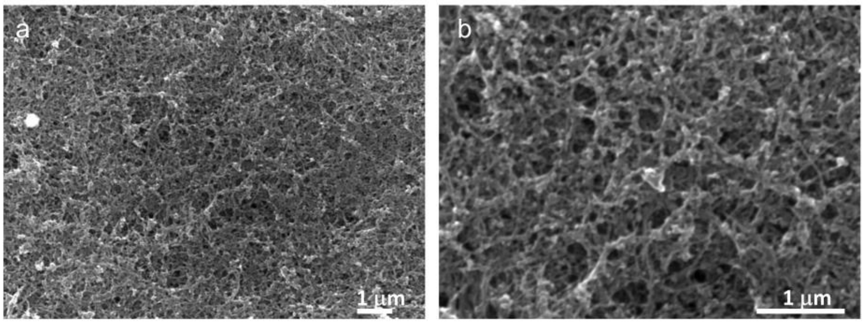

Supplementary Figure 2 | Morphology characterization of as-purchased SWCNTs. (a) and (b) SEM images of the purchased SWCNTs with 60 % purity.

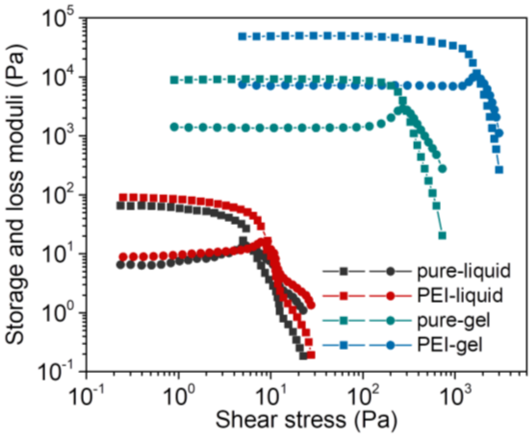

Supplementary Figure 3 | Evolution of rheological property for SWCNT/PVA. Log-log plots of shear storage and loss moduli as a function of shear stress for SWCNT/PVA compounds of varying composition and status. The cube and spherical symbols represent the storage module and loss module, respectively. The liquid composites will deform when the applied pressure exceeds the small critical values.

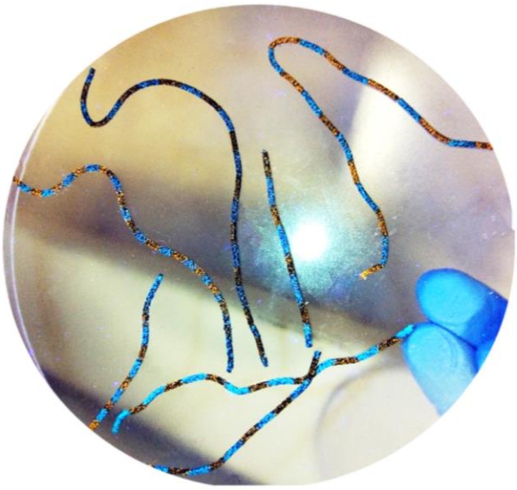

Supplementary Figure 4 | Photo for SWCNT/PVA gel. Alternating p-type (green) and n-type (yellow) SWCNT/PVA gels with different segment lengths extruded from the PTFE tube after post freezing process under UV light. To distinguish the TE types of different parts of the fiber, green and yellow ZnS UV fluorescents were beforehand mixed into the n-type (without PEI) and p-type (with PEI) SWCNT/PVA compounds, respectively. Different sizes of the core tube will result in different TE fiber size. For instance, the diameter of the inner tube of the PTFE tube is around 1.6 mm and the obtained TE fibers are around 0.8 mm in diameter after drying.

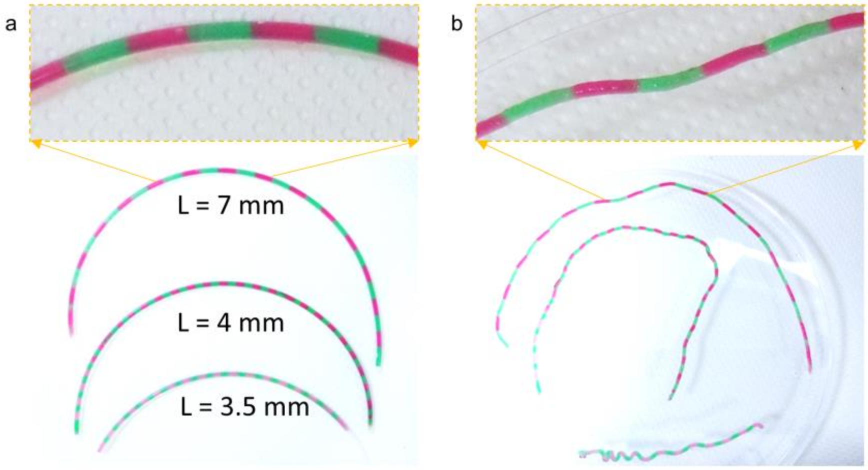

Supplementary Figure 5 | Photos for PVA gel. (a) Alternating green and red PVA gel extruded in PTFE tube. (b) Alternating green/red PVA gel extruded out from the PTFE tube after post freezing gelation process.

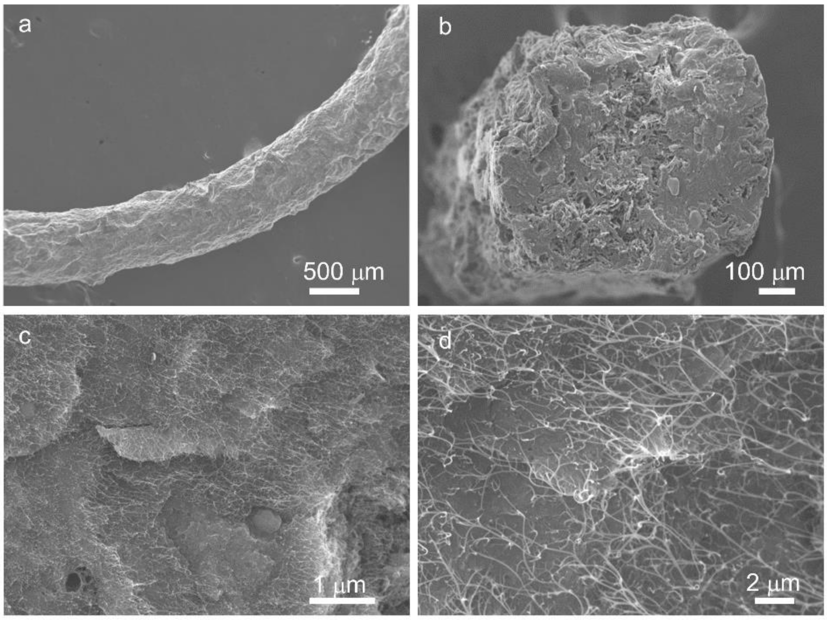

Supplementary Figure 6 | Morphology characterizations for SWCNT/PVA fiber. (a) and (b) are lateral and cross-sectional SEM images of the SWCNT/PVA fiber, respectively. Figure (c) and (d) are enlarged cross-sectional SEM images of the fiber.

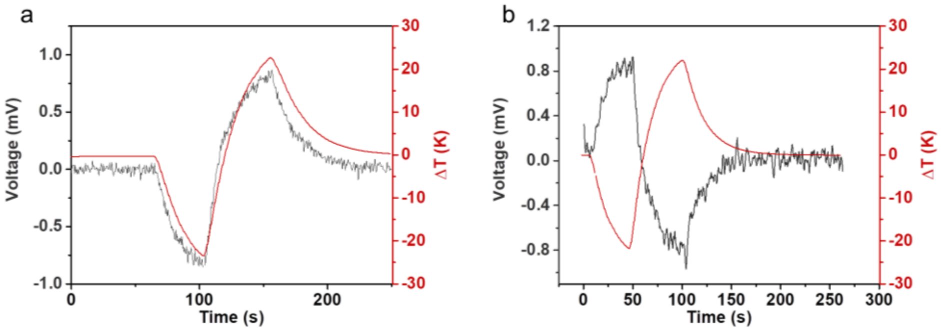

Supplementary Figure 7 | V-T curves of a single SWCNT/PVA thread. Voltage of a (a) p-type and (b) n-type SWCNT/PVA thread as the temperature difference of two terminals varies with time.

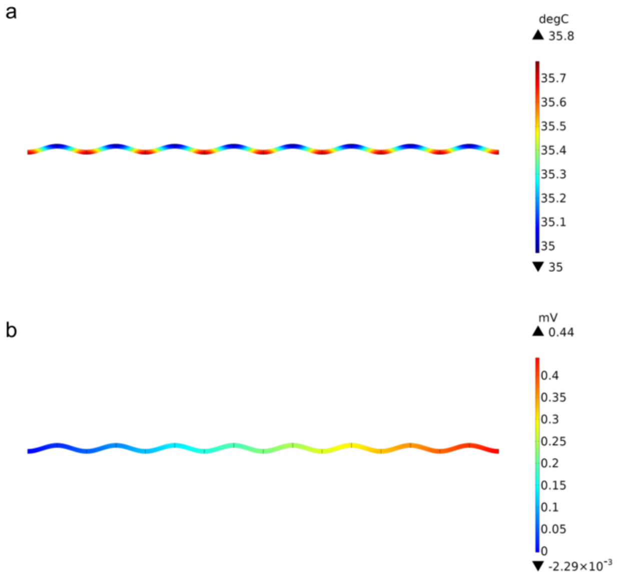

Supplementary Figure 8 | Simulation results for a single fiber. (a) Simulated temperature and (b) voltage distribution for a single fiber when placed on a substrate of 37 °C with ambient temperature of 20 °C.

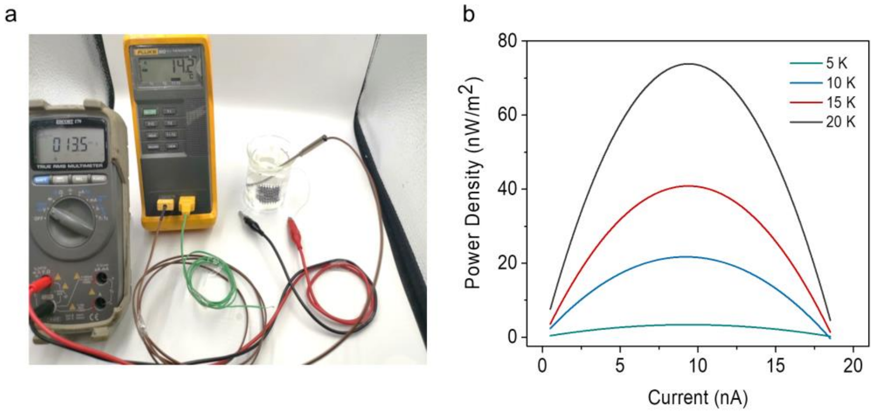

Supplementary Figure 9 | Heat energy harvesting test. (a) Heat energy harvesting test of the TE textile on a curved beaker surface with a temperature difference of ~ 14.2 °C higher than ambient. (b) Power density of the TE cloth at different temperature differences between the substrate and ambient.

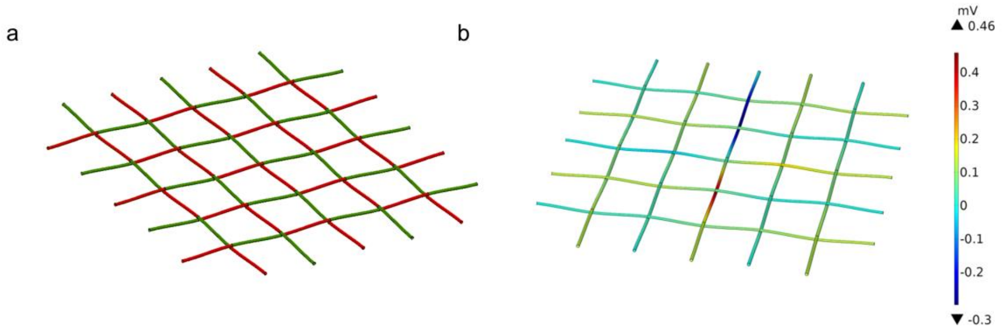

Supplementary Figure 10 | Simulation results for 5×5 pixel touch panel. (a) Configuration and simulated voltage distribution for the 5×5 pixel touch panel when a rod of 50 °C touched node (3,3).

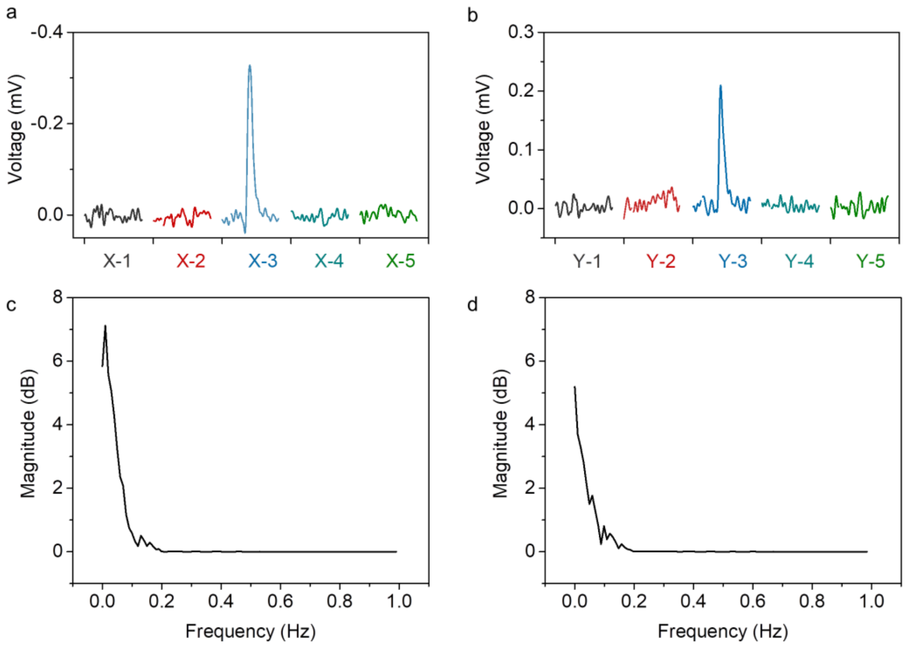

Supplementary Figure 11 | Signals for finger touching at (3,3) node. (a) and (b) are the X axis and Y axis TE voltages, respectively. (c) and (d) are the signal noise ratio (SNR) for X-3 and Y-3 fibers, respectively.

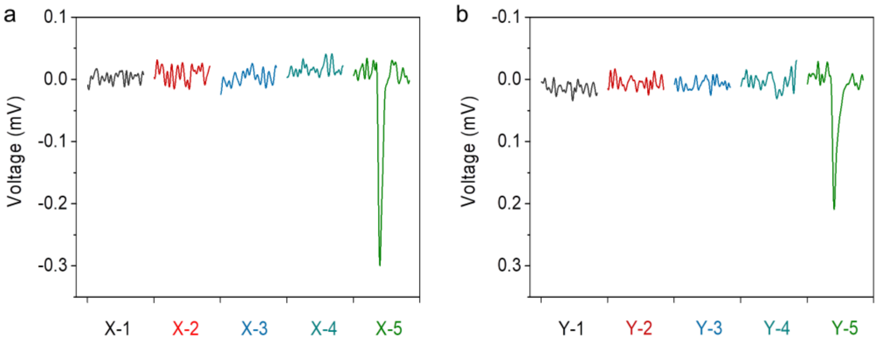

Supplementary Figure 12 | Signals for finger touching at (5,5) node. (a) and (b) are the X axis and Y axis TE voltages, respectively.

Supplementary Note 1: Thermal and electrical simulations

The thermal and electrical simulations for single fiber and pixel touch panel were performed by the finite element method. The measured thermoelectric properties of p- and n-type fibers and other parameters that utilized in the simulation were shown in Table SN1. For the single fiber numerical analysis, eight p/n pairs were series connected and supported by a substrate in the air, the hot-side and cold-side temperatures were fixed at 37 °C (substrate) and 20 °C (ambient air), as shown in Supplementary Figure 8. Besides, for the 5 × 5 pixel touch panel (Supplementary Figure 10), ten single fibers with 3 p/n pairs for each were divided into vertical and horizontal arrays with a neighbouring gap of 10 mm for plain weaving panel. The panel was put on the upper surface of the substrate and a rod touched at the node (3,3) for point-heating. The hot-side (rod) and cold-side (substrate) temperatures remained at 50 °C and 20 °C separately.

Table SN1 | Parameters of single fiber and panel for simulation

Parameter / Units	Composition	Dimension $d \times l$ (mm)	Thermal conductivity κ (W m^{-1} K^{-1})	Electrical conductivity σ (S/m)	Seebeck coefficient S (μV K^{-1})
p-type segment	SWCNT/PVA	0.5 × 10	0.15	7.7	39.5
n-type segment	SWCNT/PVA	0.5 × 10	0.15	1.11	-46
Substrate*	Acrylic	5 × 160 × 5	0.18	/	/
Substrate**	Acrylic	10 × 60 × 60	0.18	/	/
Rod	Fe	2.5 × 50	80.2	1.02E7	/

* and ** stand for the supported substrate (thickness × length × width) used for single fiber and touch panel simulation respectively.

Supplementary Note 2: Controlling modules for the demonstrations

The electrical signal of the fibers in the demonstrations were acquired using a Raspberry Pi equipped with a 24 bit ADC expansion module (ADS1256). In the TE textile touch panel, the individual vertical and horizontal line fiber electrical signals were multiplexed using a 16 channel analog/digital multiplexing module (SparkFun CD74HC4067) (circuit diagram is shown in Supplementary Note 2 Fig. SN2a). A Python script was written to multiply each vertical and horizontal fiber absolute voltage and visualized as a heat map. In the TE fiber light communication, a Python script was written to process the voltages from the fiber of each cubic face, emitting a PWM signal that correspond to the color of the face with the light being shone (circuit diagram is shown in Supplementary Note 2 Fig. SN2b). For the hot/cold perception glove and phototaxis wrist band, the Raspberry Pi provided the PWM signals to drive a 16 channel 12 bit PWM/servo driver (PCA9685) to actuate the robotic arm (circuit diagram is shown in Supplementary Note 2 Fig. SN2c).

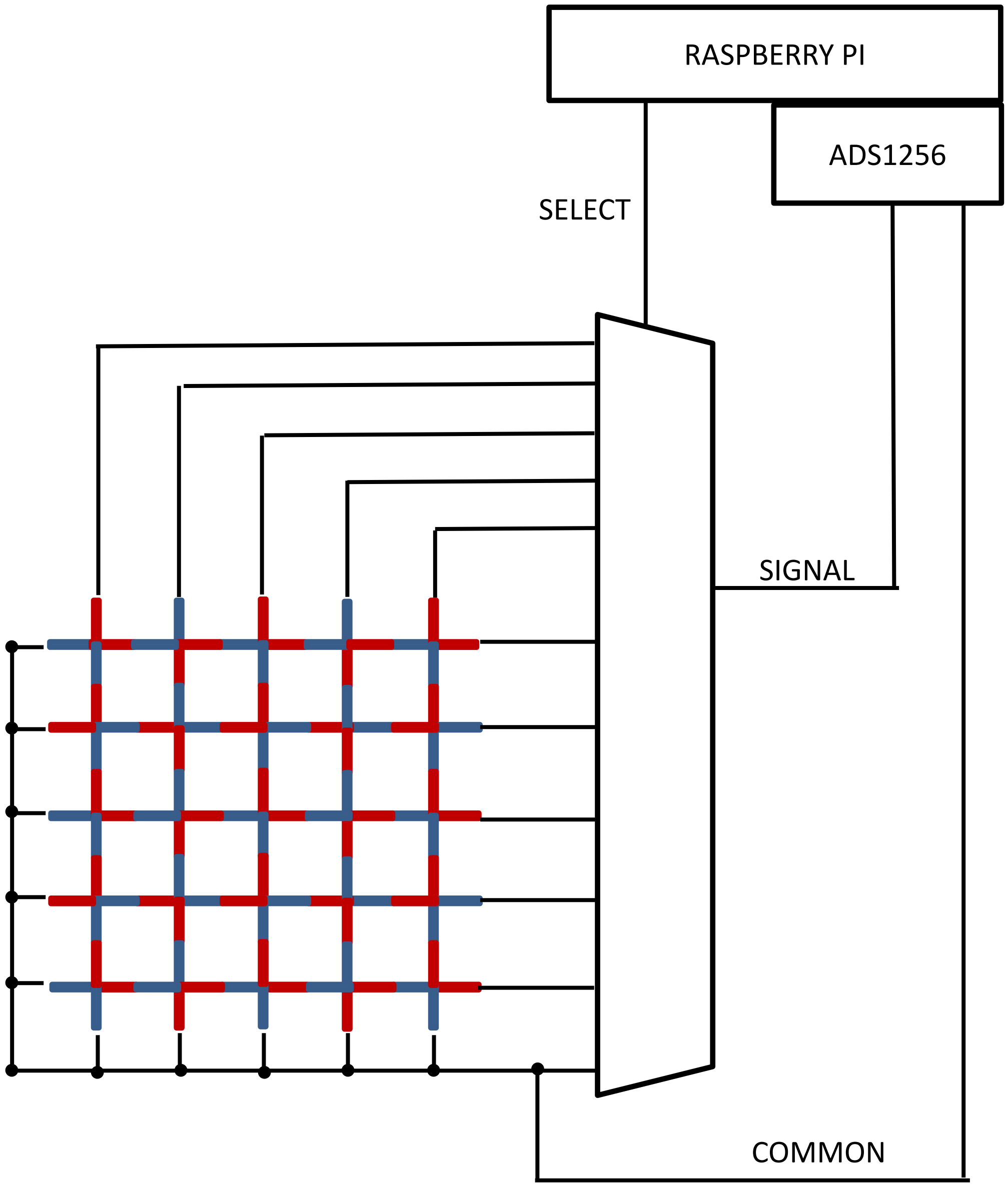

Supplementary Note 2 Fig. SN2a | Circuit diagram for TE textile touch panel.

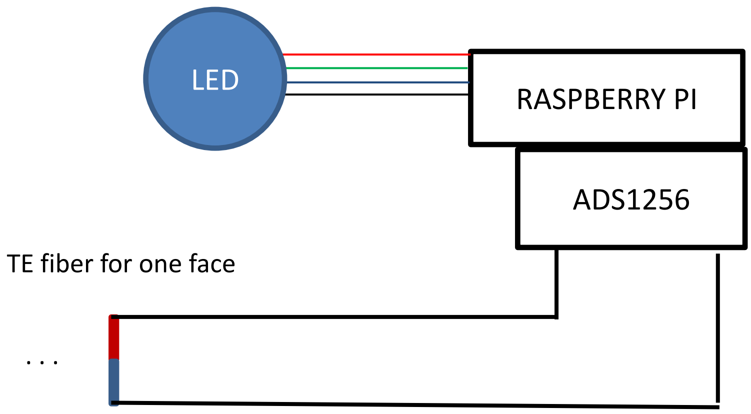

Supplementary Note 2 Fig. SN2b | Circuit diagram for color cube.

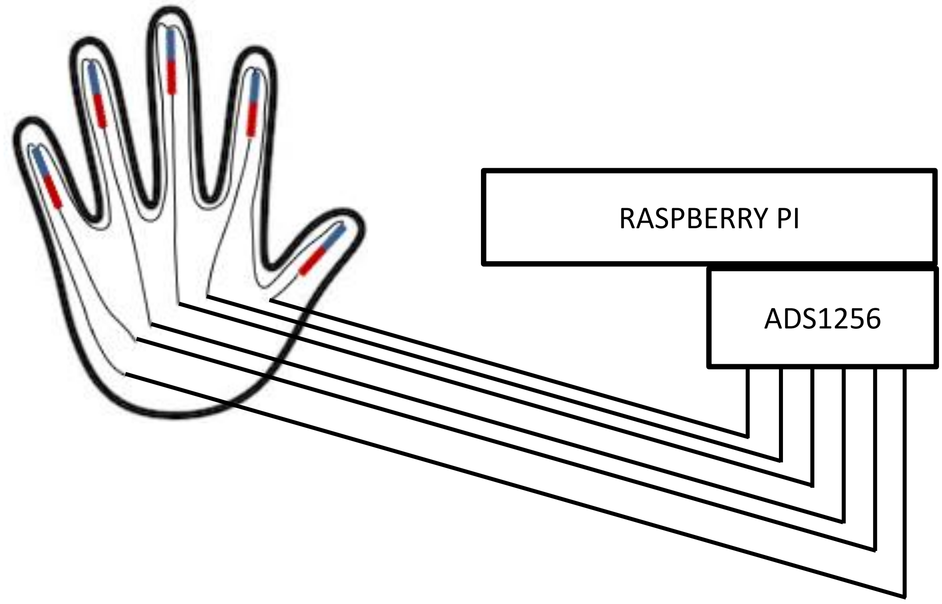

Supplementary Note 2 Fig. SN2c | Connection diagram for hot/cold perception glove.
