# Supplementary material for: Scalable thermoelectric fibers for multifunctional textile-electronics
Source: Nat Commun. 2020 Nov 26;11:6006. doi: 10.1038/s41467-020-19867-7 (PMC7693281; doi:10.1038/s41467-020-19867-7)
Supplement: Supplementary file 7 — Description of Additional Supplementary Files [file 41467_2020_19867_MOESM7_ESM.pdf]

:
File Name: Supplementary Movie 1
Description: Alternating extrude-segment process.

File Name: Supplementary Movie 2
Description: Stability test of TE fabric with washing in water and rubbing.

File Name: Supplementary Movie 3
Description: Handwriting ‘NUS’ alphabet input.

File Name: Supplementary Movie 4
Description: Light communication device to perceive the incident light orientation.

File Name: Supplementary Movie 5
Description: Feedback control of robot arm wearing TE garments including reflex of hot/cold subject and phototaxis.
